# Supplementary material for: Synthesis and Biological Evaluation of Potential Oncoimmunomodulator Agents
Source: Int J Mol Sci. 2023 Jan 30;24(3):2614. doi: 10.3390/ijms24032614 (PMC9917184; doi:10.3390/ijms24032614)
Supplement: Supplementary file 1 [file ijms-24-02614-s001.zip › ijms-2147175-supplementary.pdf]

Title Synthesis and Biological Evaluation of Potential Oncoimmunomodulator Agents

Authors Raquel Gil-Edo[1], Sara Espejo[1], Eva Falomir[1*] and Miguel Carda[1]

Supporting Information

Contents:

Graphical NMR spectra.. - 7 -

Graphical NMR spectra

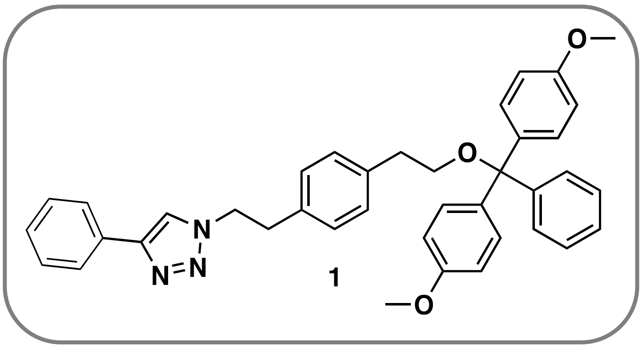

1

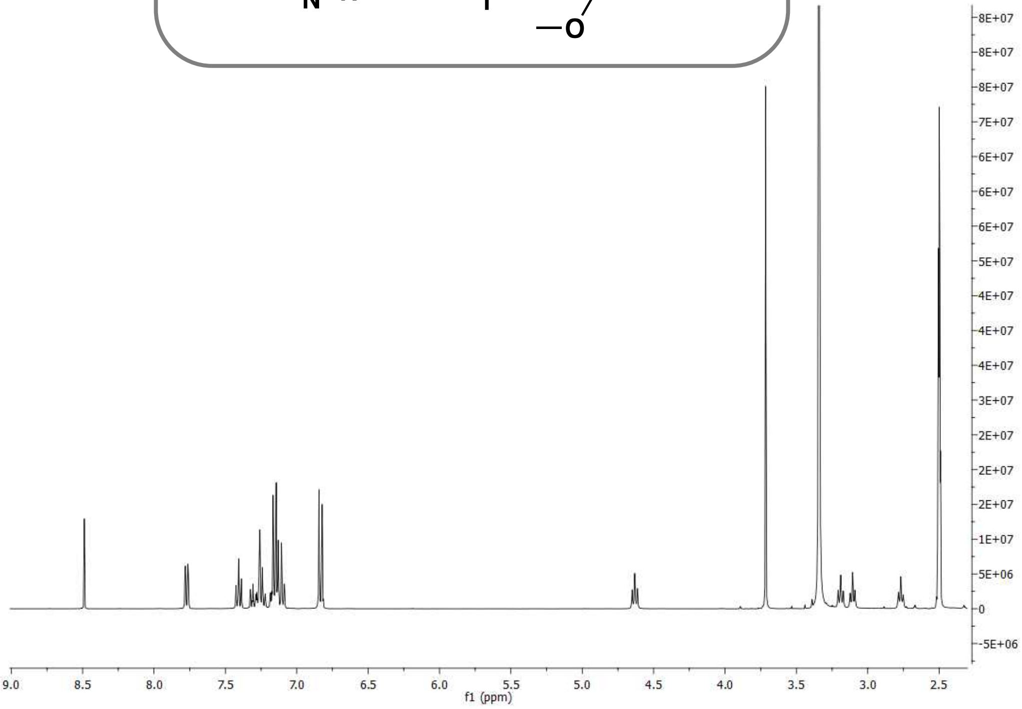

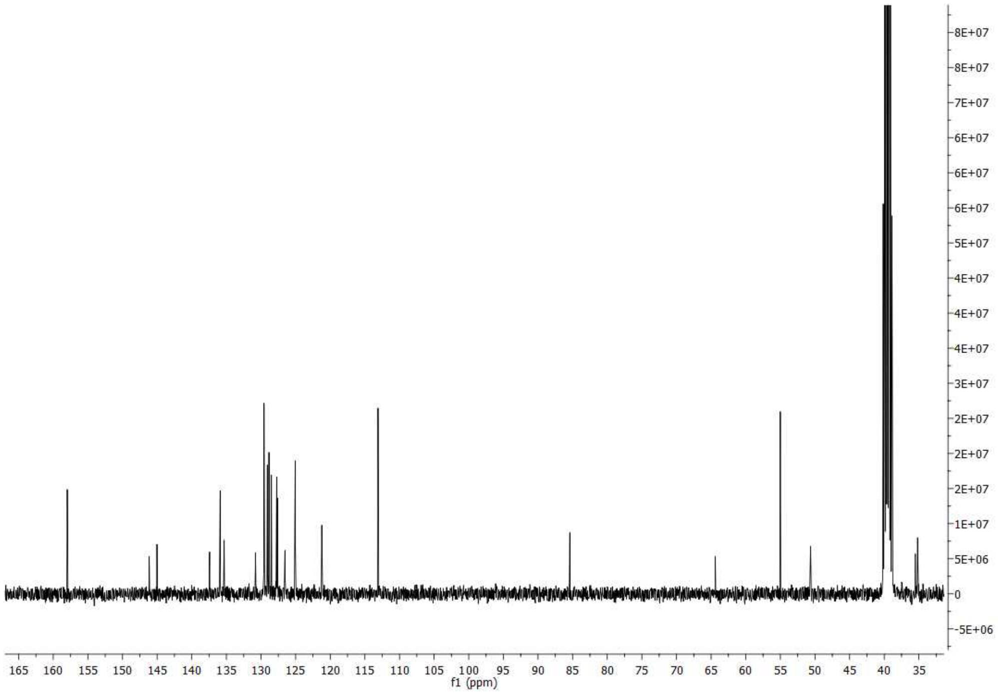

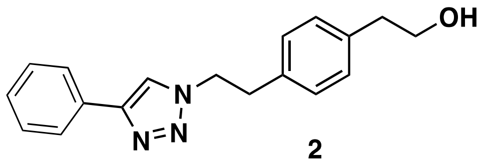
N
N
N
OH

2

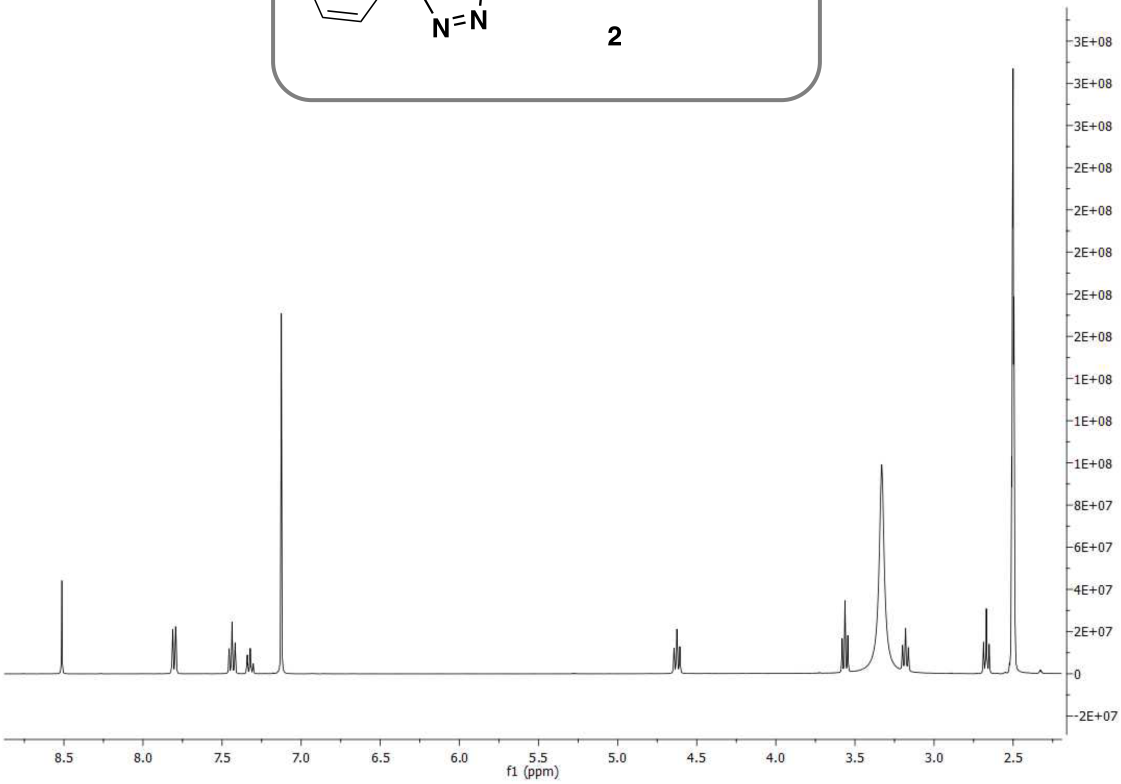
8.5 8.0 7.5 7.0 6.5 6.0 5.5 5.0 4.5 4.0 3.5 3.0 2.5
f1 (ppm)
3E+08
3E+08
3E+08
2E+08
2E+08
2E+08
2E+08
2E+08
1E+08
1E+08
1E+08
8E+07
6E+07
4E+07
2E+07
0
-2E+07

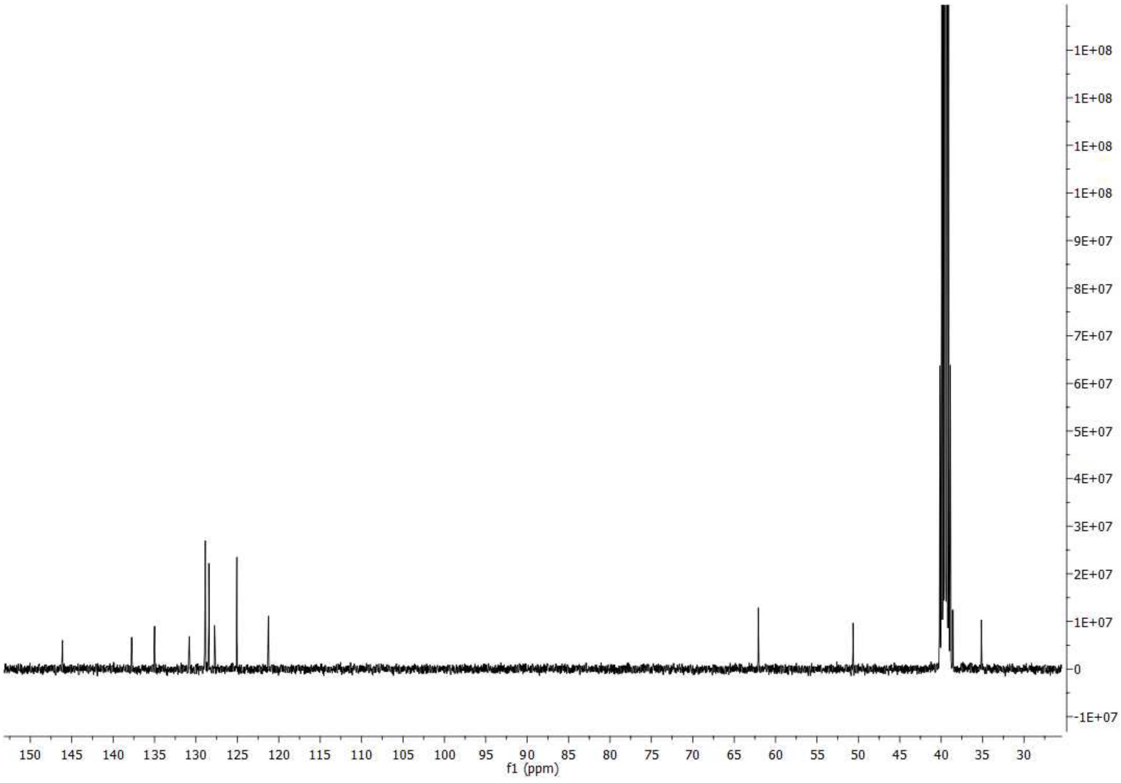
150 145 140 135 130 125 120 115 110 105 100 95 90 85 80 75 70 65 60 55 50 45 40 35 30
f1 (ppm)
1E+08
1E+08
1E+08
1E+08
9E+07
8E+07
7E+07
6E+07
5E+07
4E+07
3E+07
2E+07
1E+07
0
-1E+07

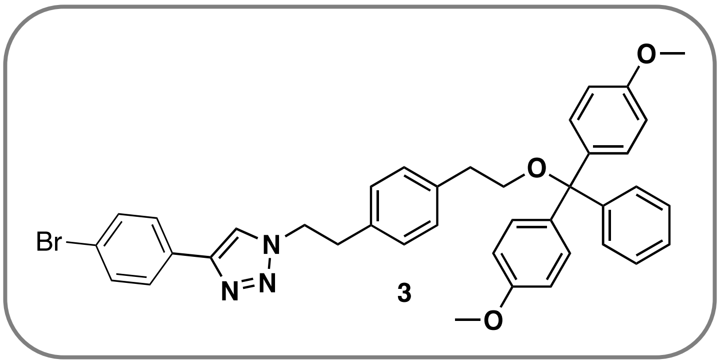
Br
N
N=N
O
O
O
3

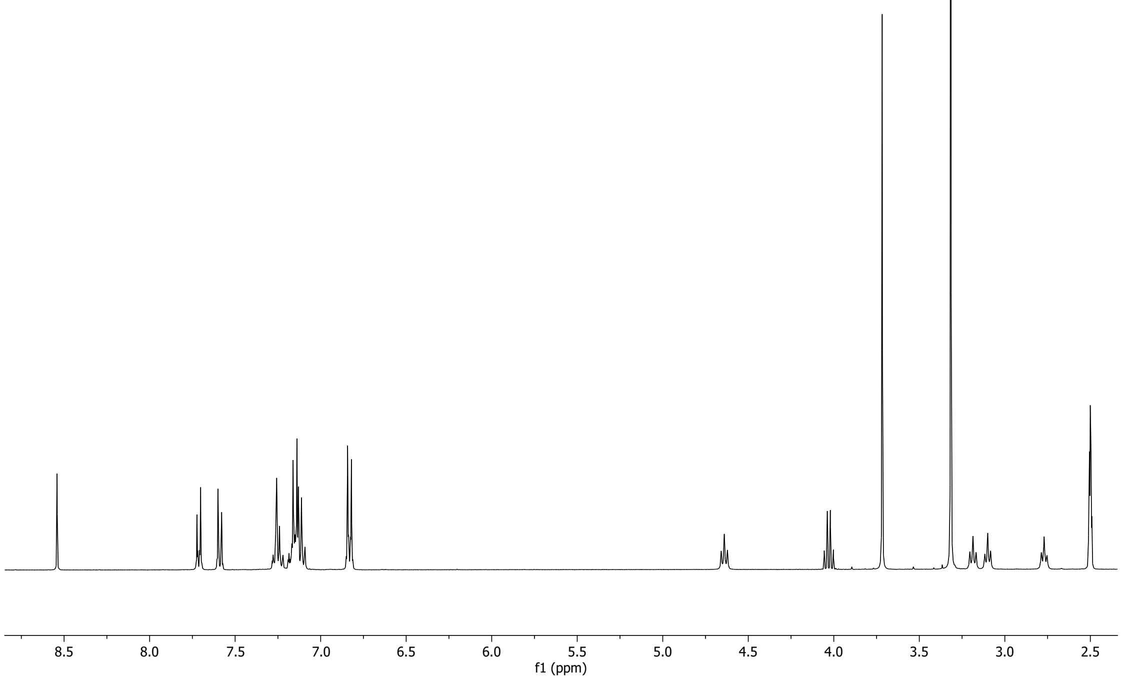
8.5 8.0 7.5 7.0 6.5 6.0 5.5 5.0 4.5 4.0 3.5 3.0 2.5
f1 (ppm)

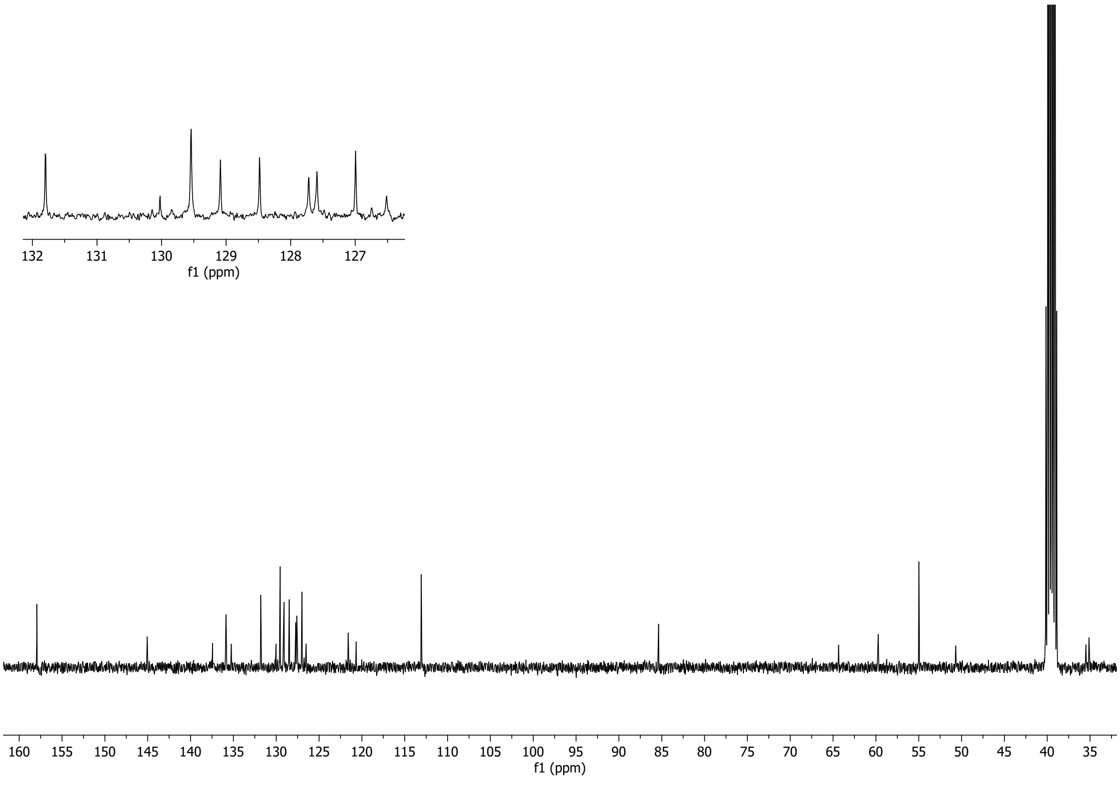
132 131 130 129 128 127
f1 (ppm)
160 155 150 145 140 135 130 125 120 115 110 105 100 95 90 85 80 75 70 65 60 55 50 45 40 35
f1 (ppm)

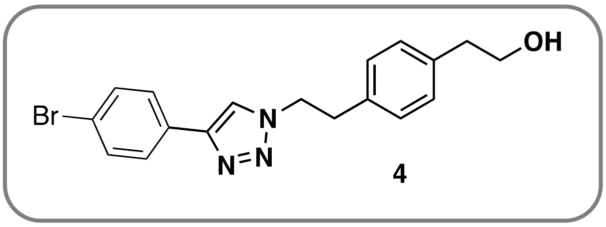
Br
N
N=N
OH
4

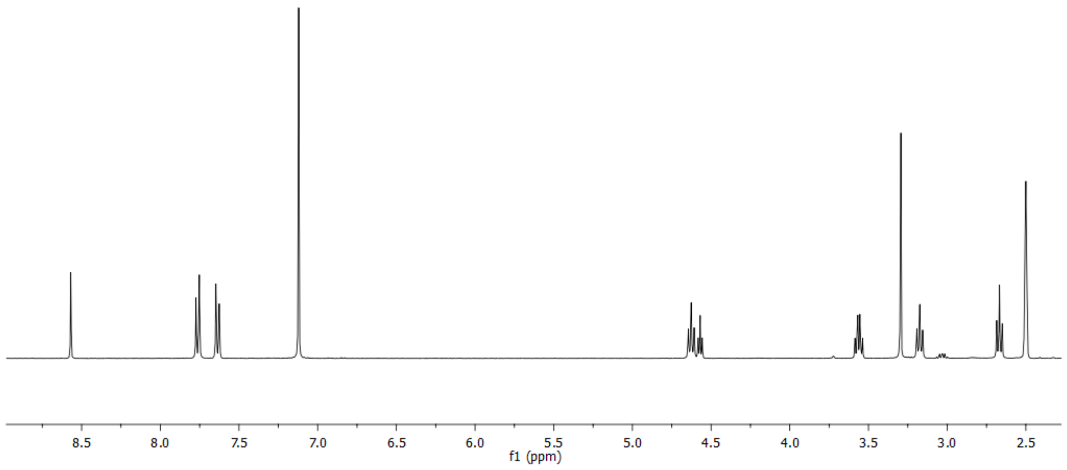
8.5 8.0 7.5 7.0 6.5 6.0 5.5 5.0 4.5 4.0 3.5 3.0 2.5
f1 (ppm)

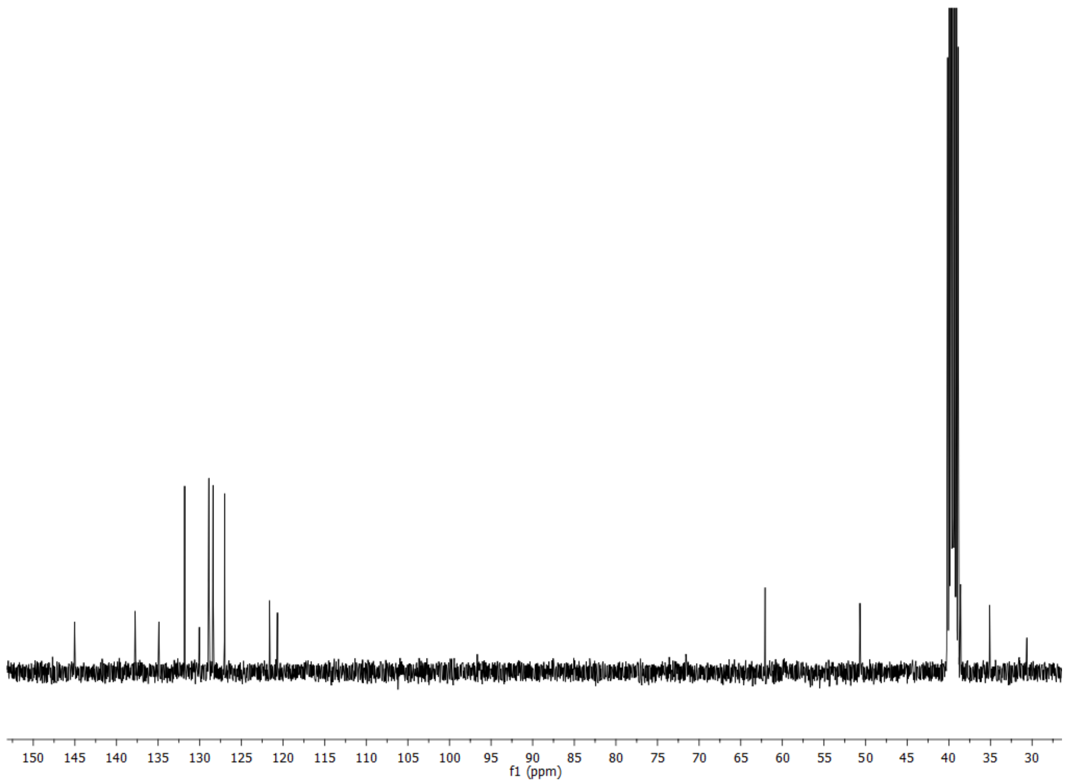
150 145 140 135 130 125 120 115 110 105 100 95 90 85 80 75 70 65 60 55 50 45 40 35 30
f1 (ppm)

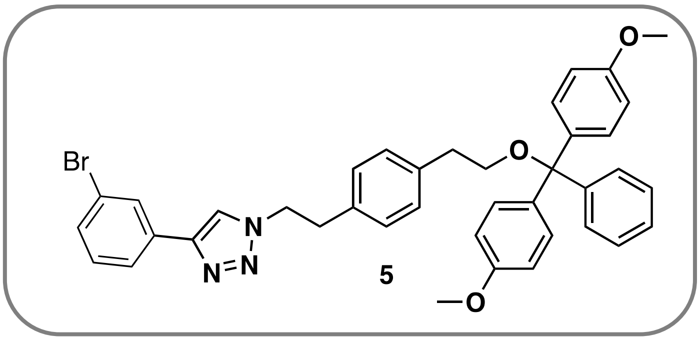
Br
N
N=N
O
O
O
5

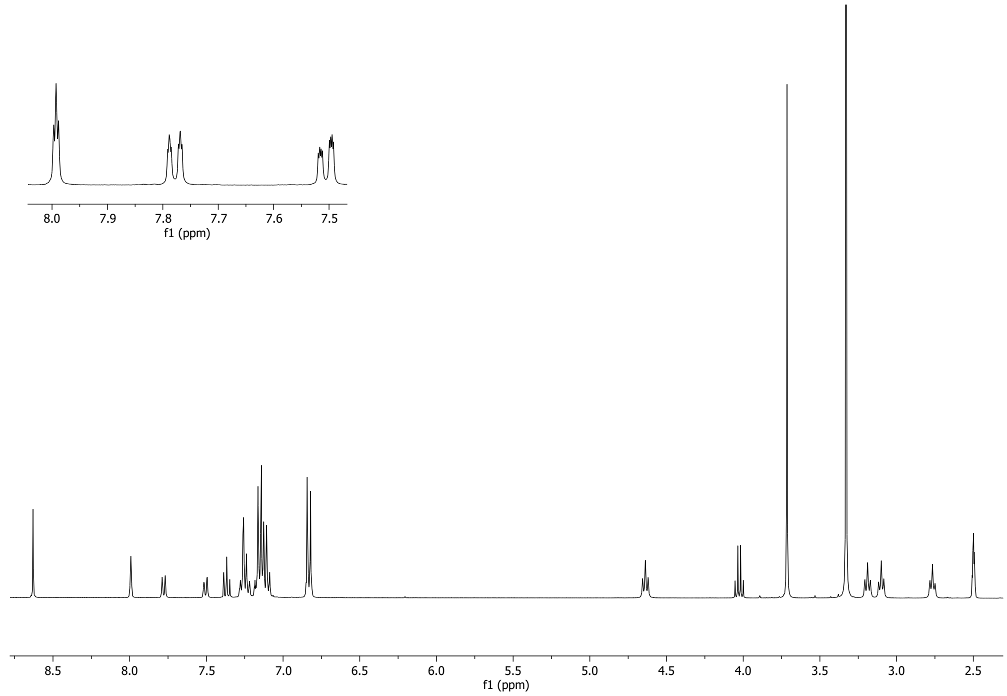
f1 (ppm)
f1 (ppm)

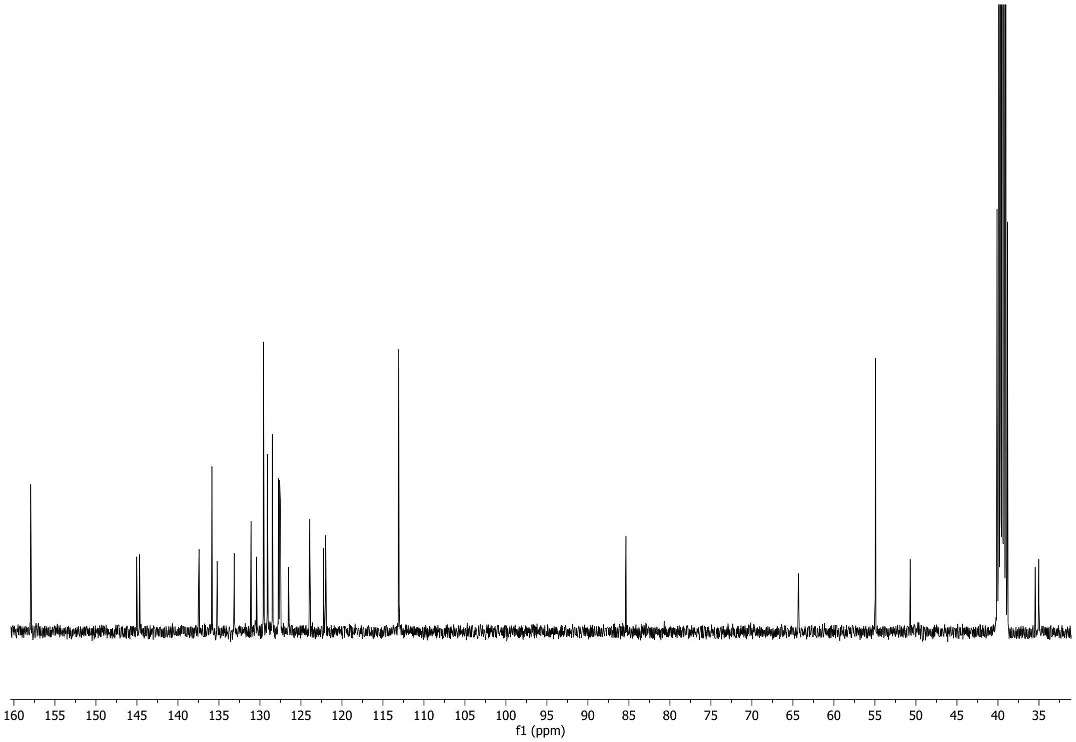
f1 (ppm)

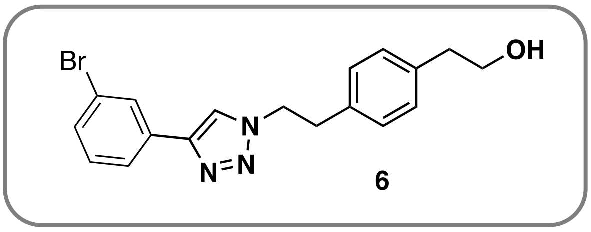
Br
N
N=N
OH
6

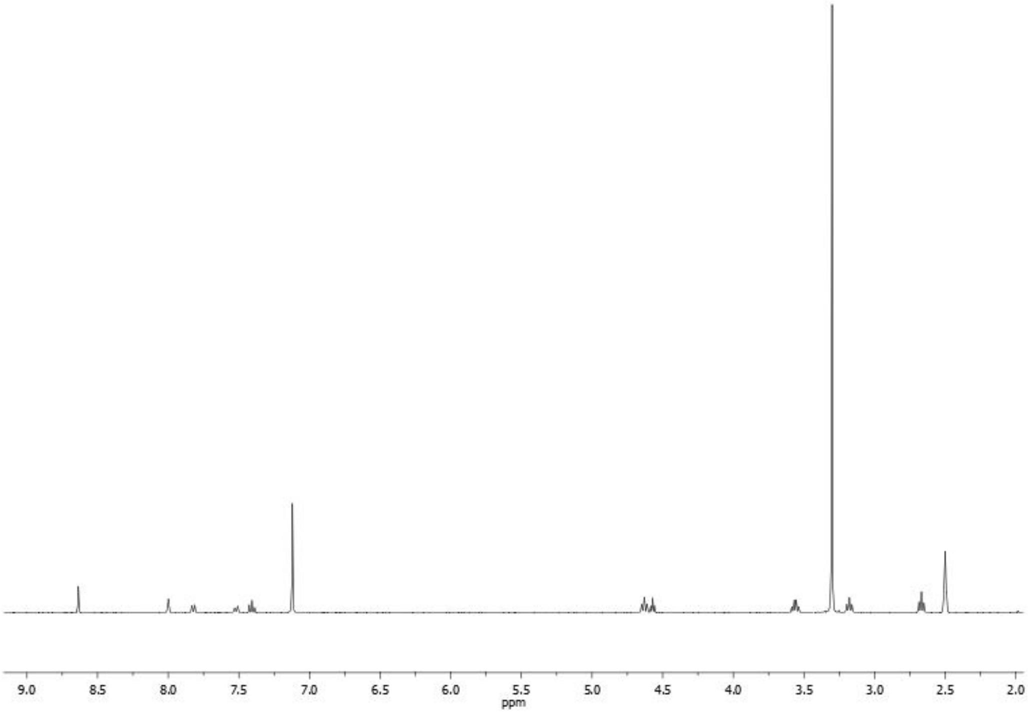
9.0 8.5 8.0 7.5 7.0 6.5 6.0 5.5 5.0 4.5 4.0 3.5 3.0 2.5 2.0
ppm

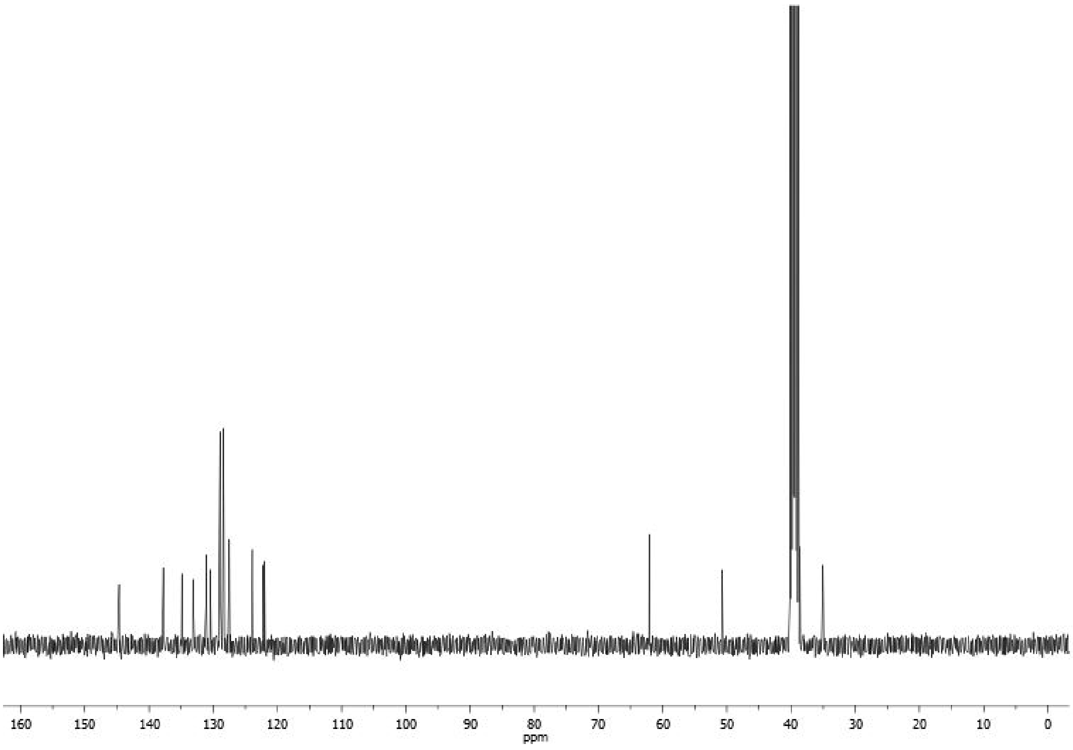
160 150 140 130 120 110 100 90 80 70 60 50 40 30 20 10 0
ppm

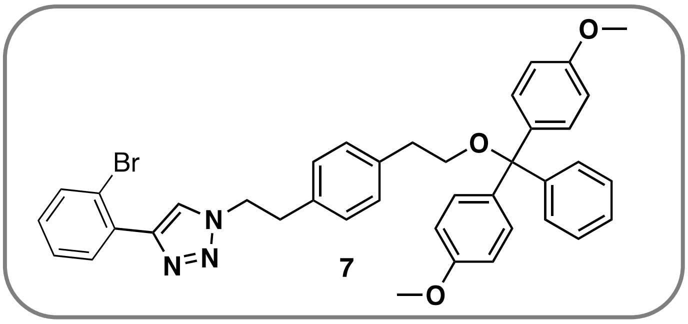
Br
N
N
N
O
O
O
7

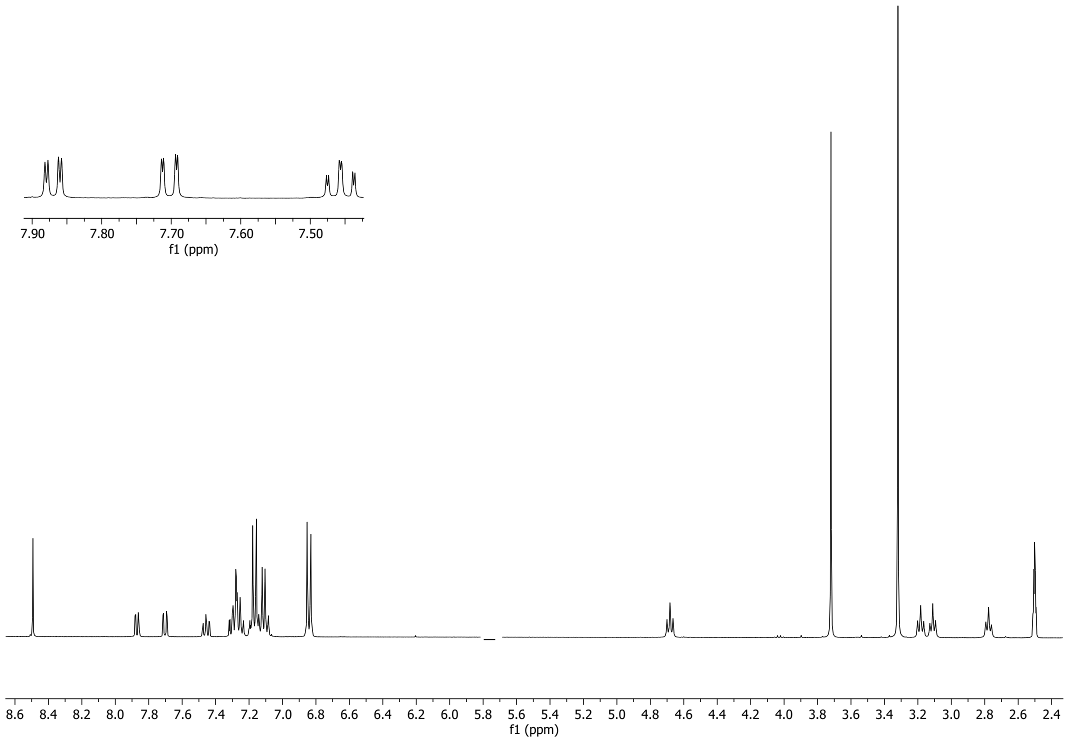
7.90 7.80 7.70 7.60 7.50
f1 (ppm)
8.6 8.4 8.2 8.0 7.8 7.6 7.4 7.2 7.0 6.8 6.6 6.4 6.2 6.0 5.8 5.6 5.4 5.2 5.0 4.8 4.6 4.4 4.2 4.0 3.8 3.6 3.4 3.2 3.0 2.8 2.6 2.4
f1 (ppm)

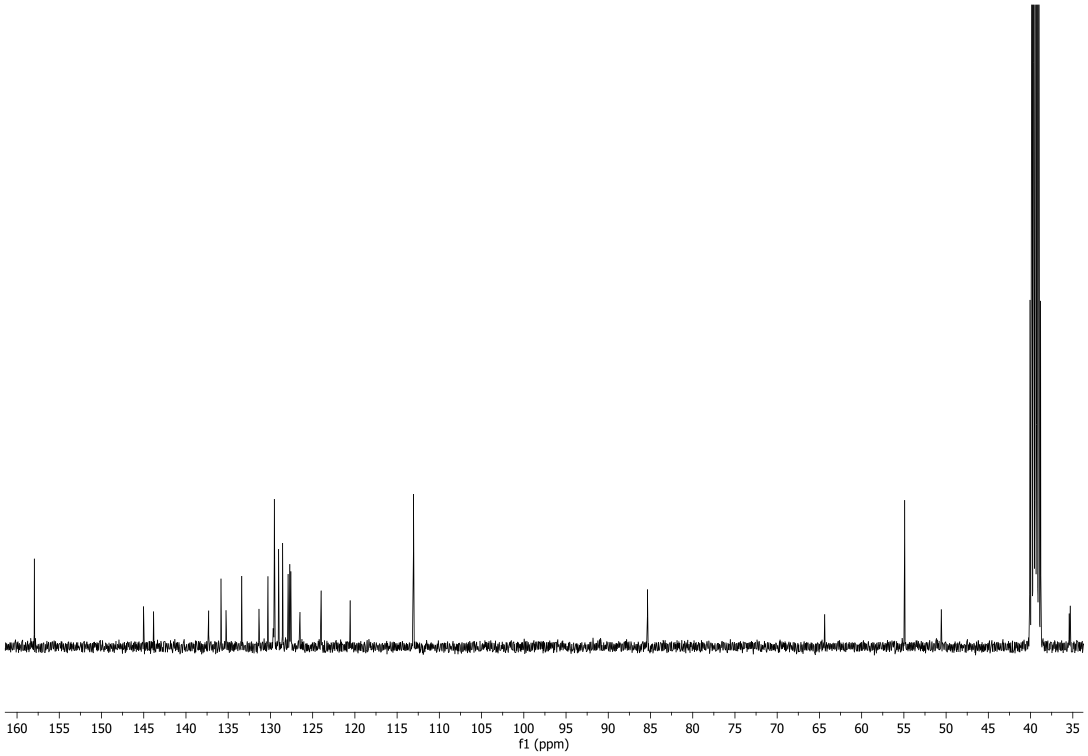
160 155 150 145 140 135 130 125 120 115 110 105 100 95 90 85 80 75 70 65 60 55 50 45 40 35
f1 (ppm)

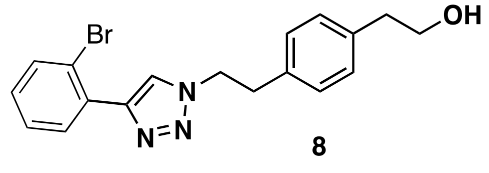

8

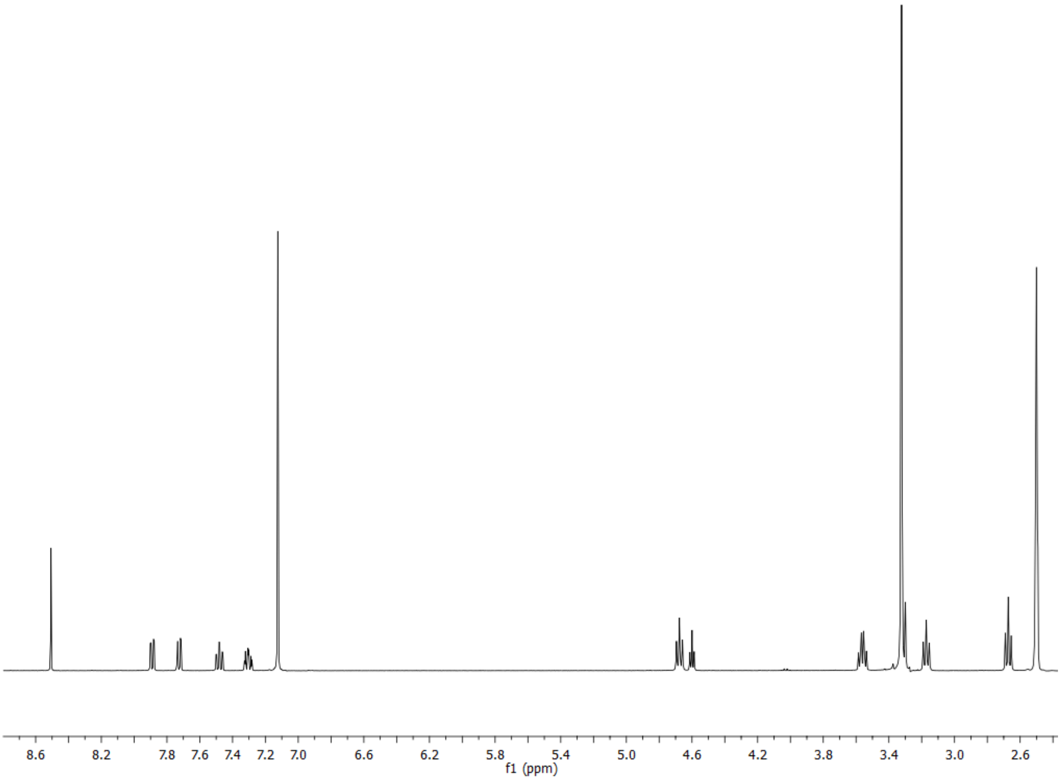

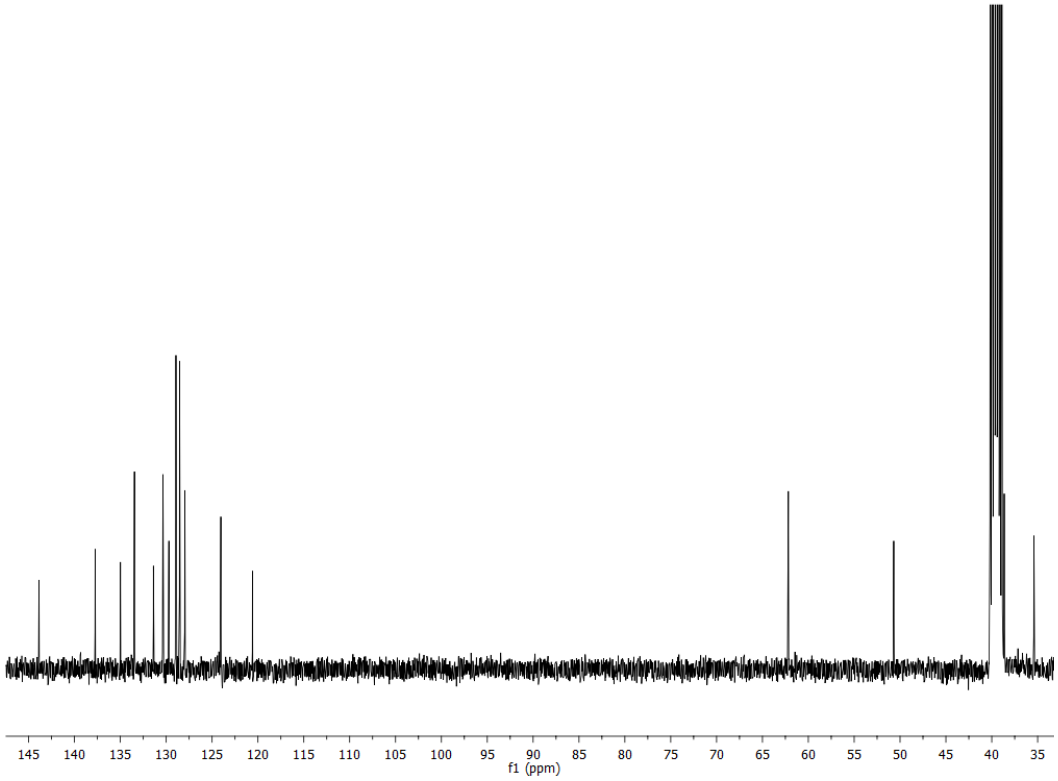
145 140 135 130 125 120 115 110 105 100 95 90 85 80 75 70 65 60 55 50 45 40 35
f1 (ppm)

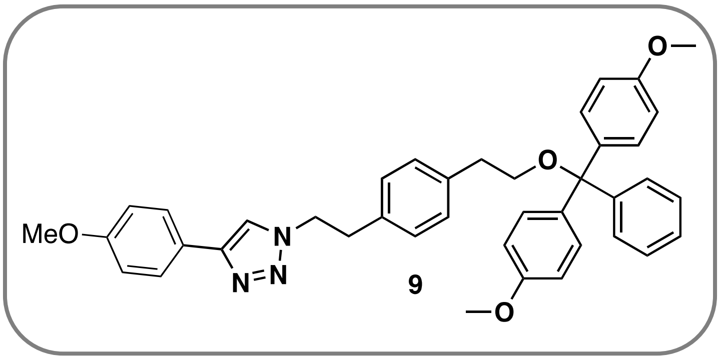
MeO
N
N=N
O
9

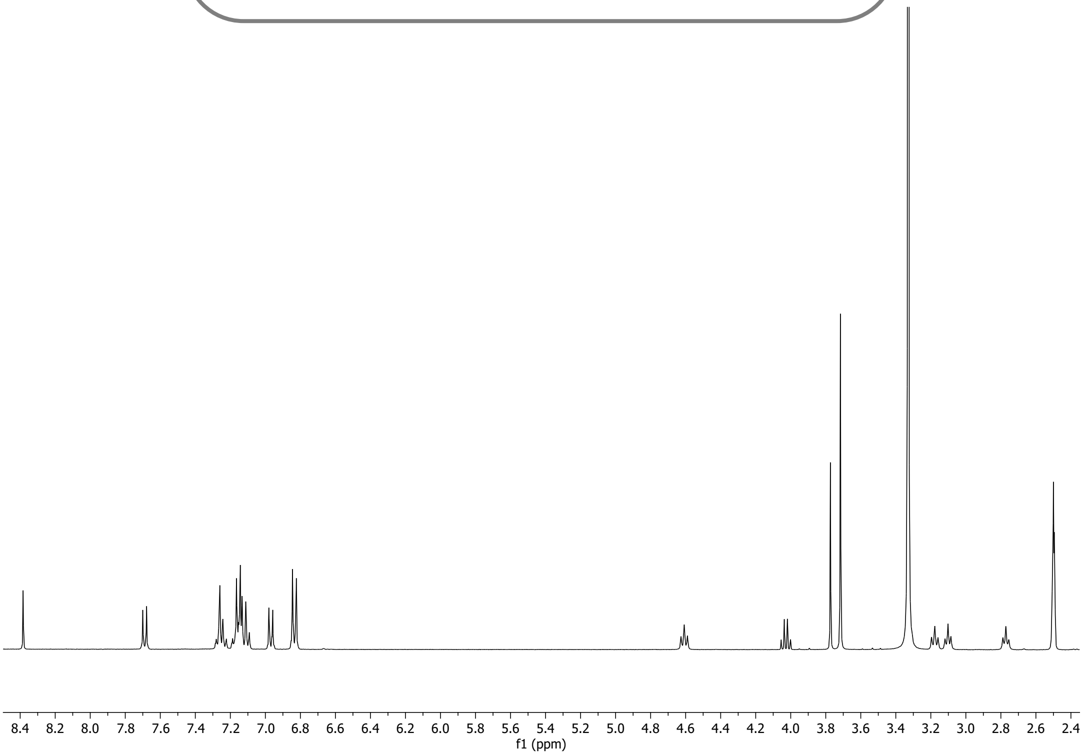
8.4 8.2 8.0 7.8 7.6 7.4 7.2 7.0 6.8 6.6 6.4 6.2 6.0 5.8 5.6 5.4 5.2 5.0 4.8 4.6 4.4 4.2 4.0 3.8 3.6 3.4 3.2 3.0 2.8 2.6 2.4
f1 (ppm)

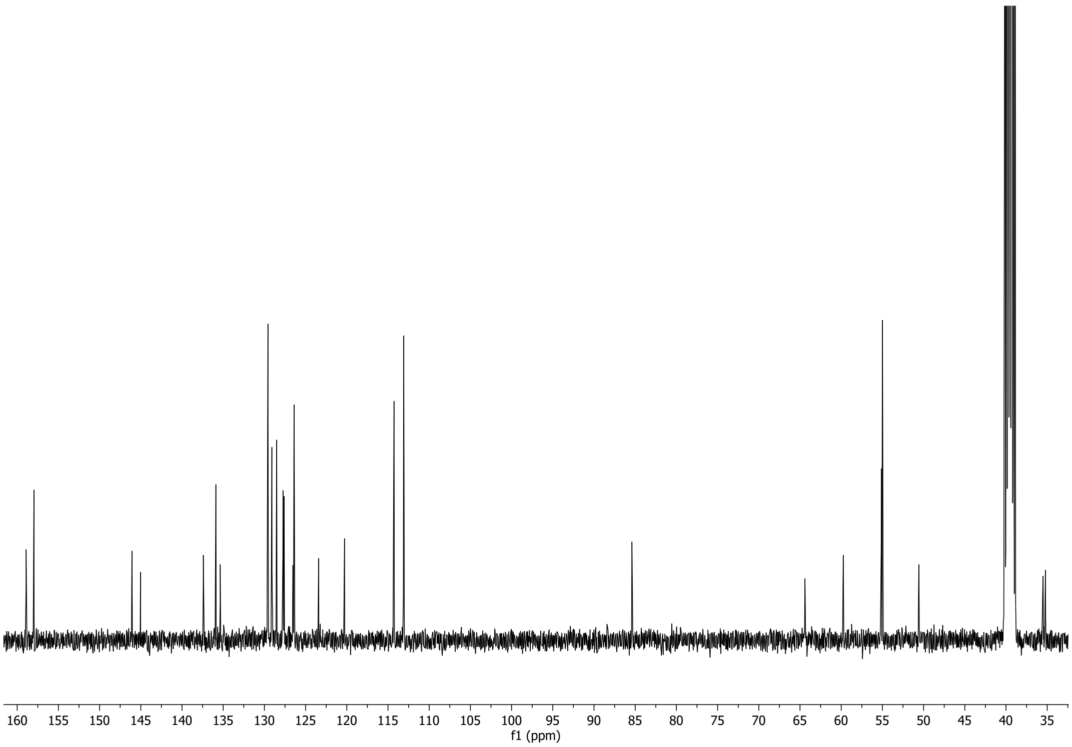
160 155 150 145 140 135 130 125 120 115 110 105 100 95 90 85 80 75 70 65 60 55 50 45 40 35
f1 (ppm)

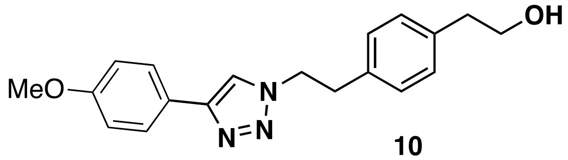
MeO
N
N=N
OH

10

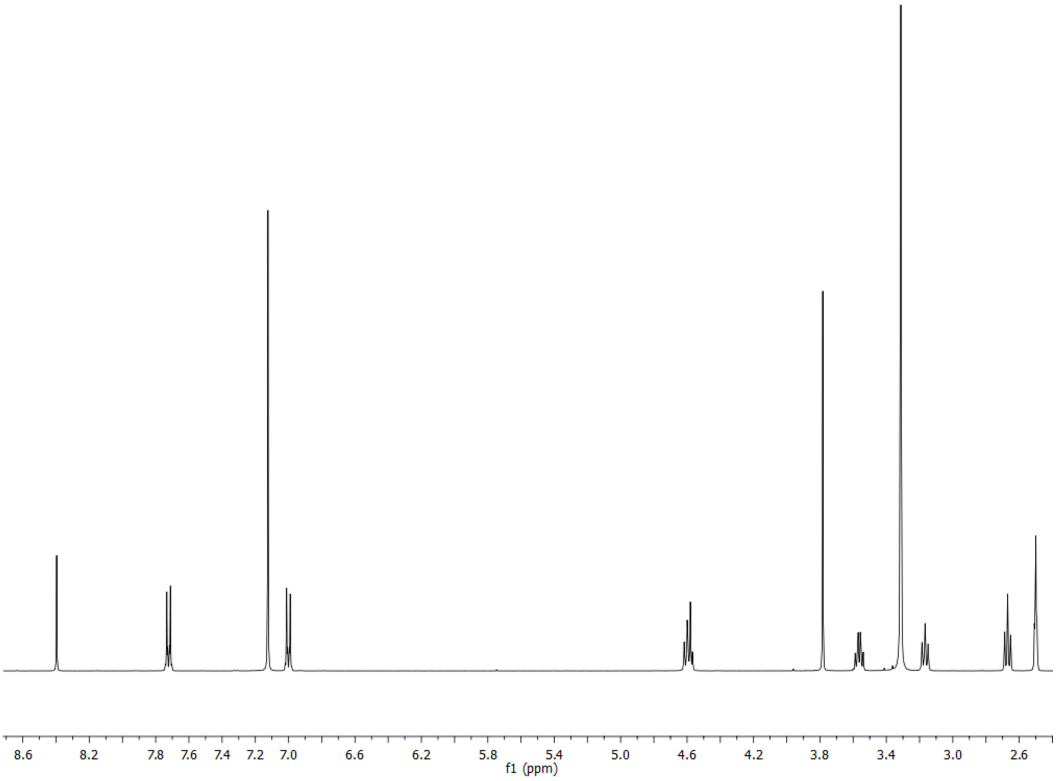
8.6 8.2 7.8 7.6 7.4 7.2 7.0 6.6 6.2 5.8 5.4 5.0 4.6 4.2 3.8 3.4 3.0 2.6
f1 (ppm)

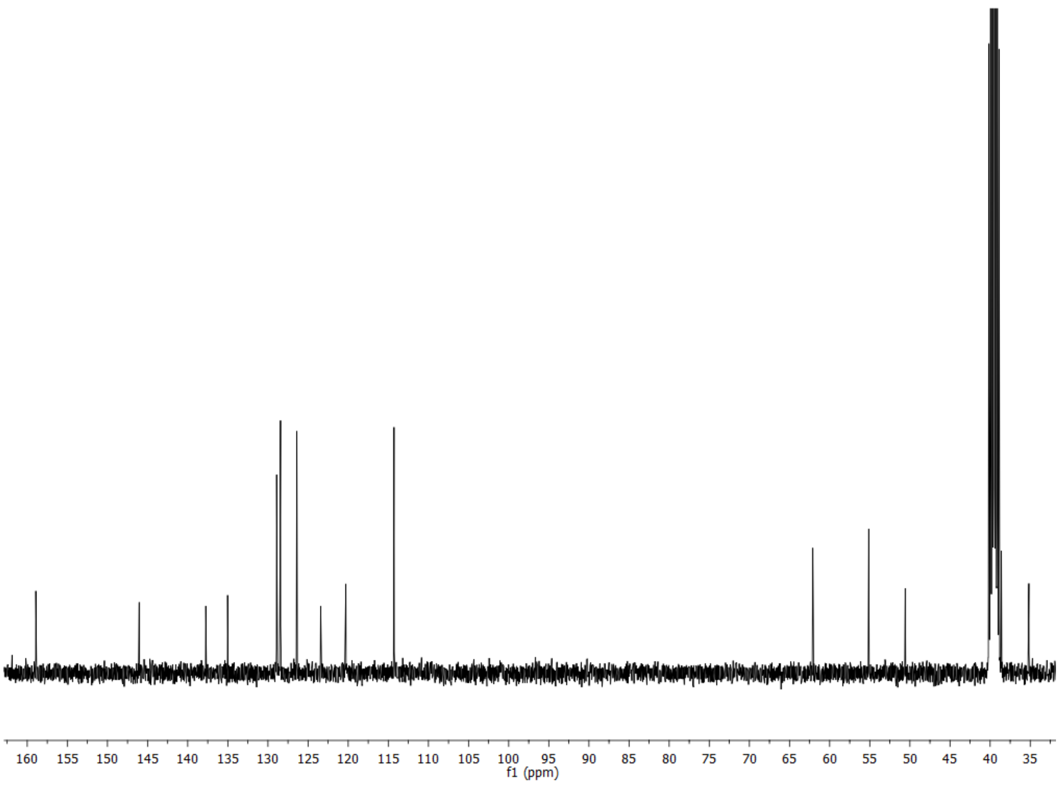
160 155 150 145 140 135 130 125 120 115 110 105 100 95 90 85 80 75 70 65 60 55 50 45 40 35
f1 (ppm)

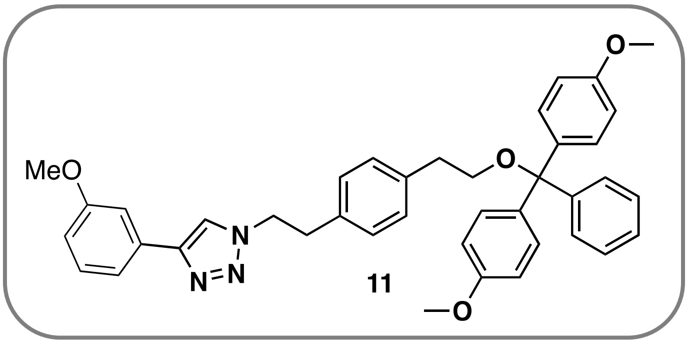
MeO
N
N=N
O
11

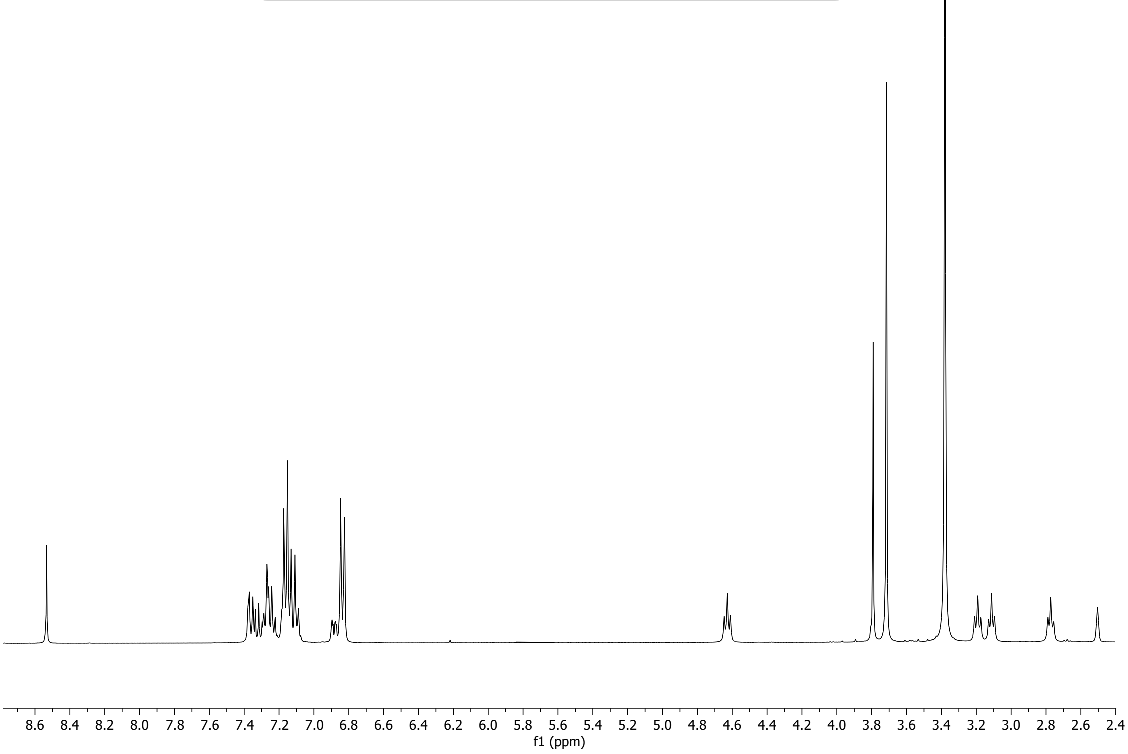
8.6 8.4 8.2 8.0 7.8 7.6 7.4 7.2 7.0 6.8 6.6 6.4 6.2 6.0 5.8 5.6 5.4 5.2 5.0 4.8 4.6 4.4 4.2 4.0 3.8 3.6 3.4 3.2 3.0 2.8 2.6 2.4
f1 (ppm)

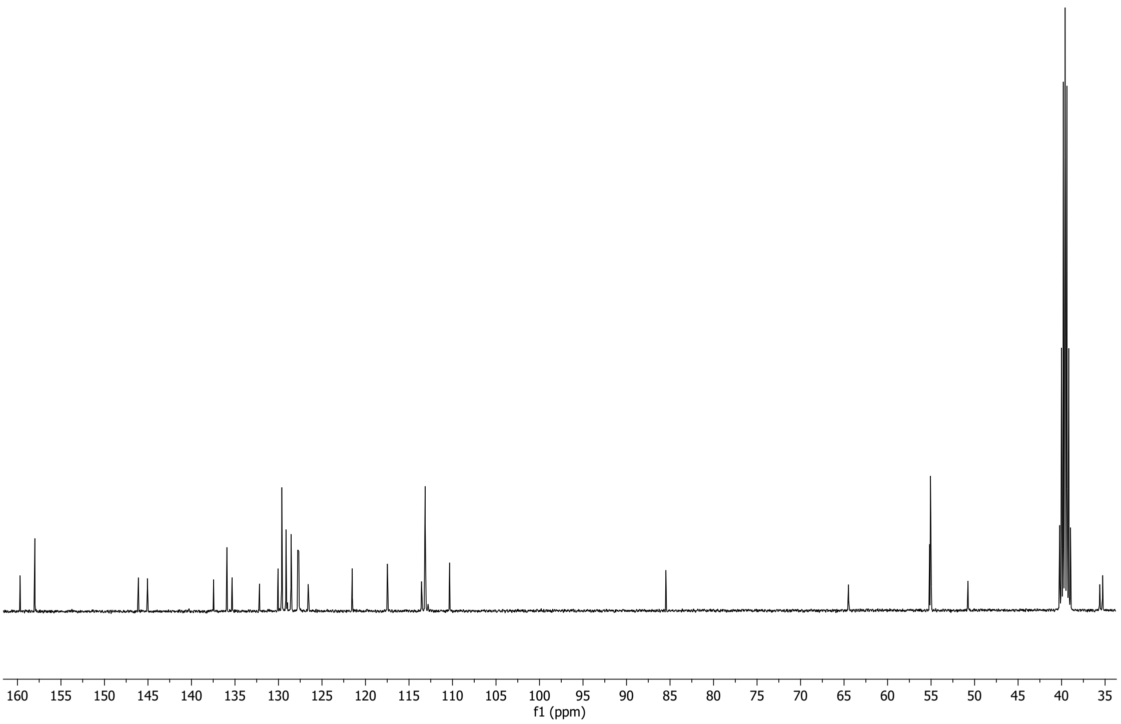
160
155
150
145
140
135
130
125
120
115
110
105
100
95
90
85
80
75
70
65
60
55
50
45
40
35
f1 (ppm)

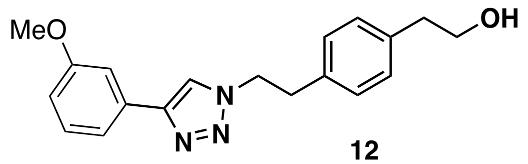
MeO
N
N
N
OH

12

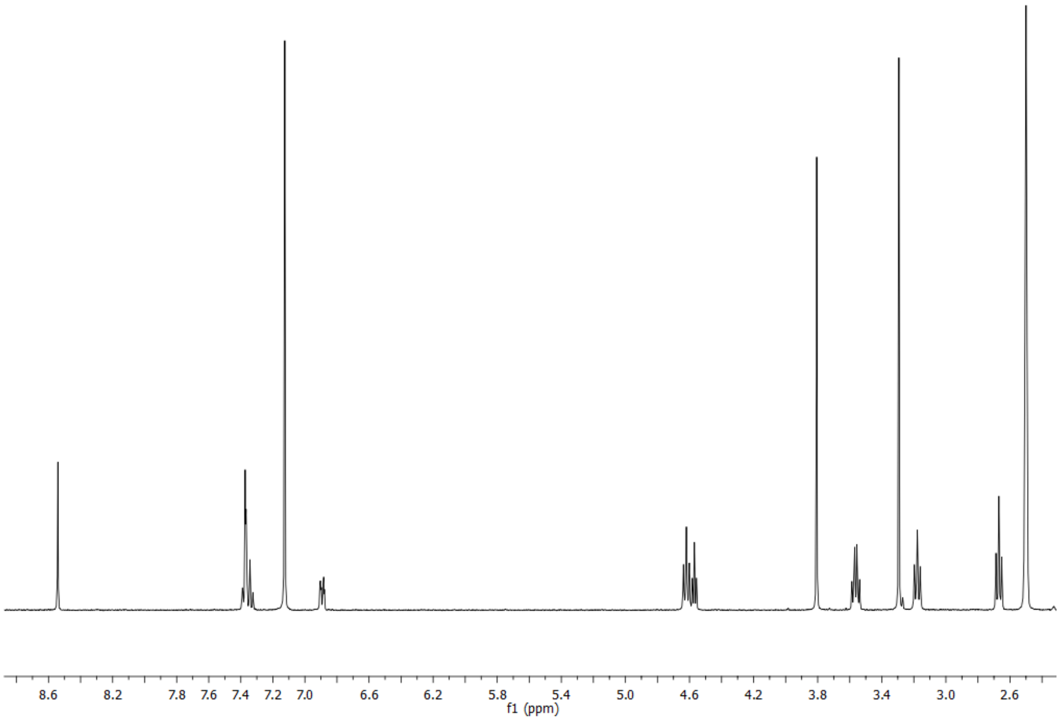
8.6
8.2
7.8
7.6
7.4
7.2
7.0
6.6
6.2
5.8
5.4
5.0
4.6
4.2
3.8
3.4
3.0
2.6
f1 (ppm)

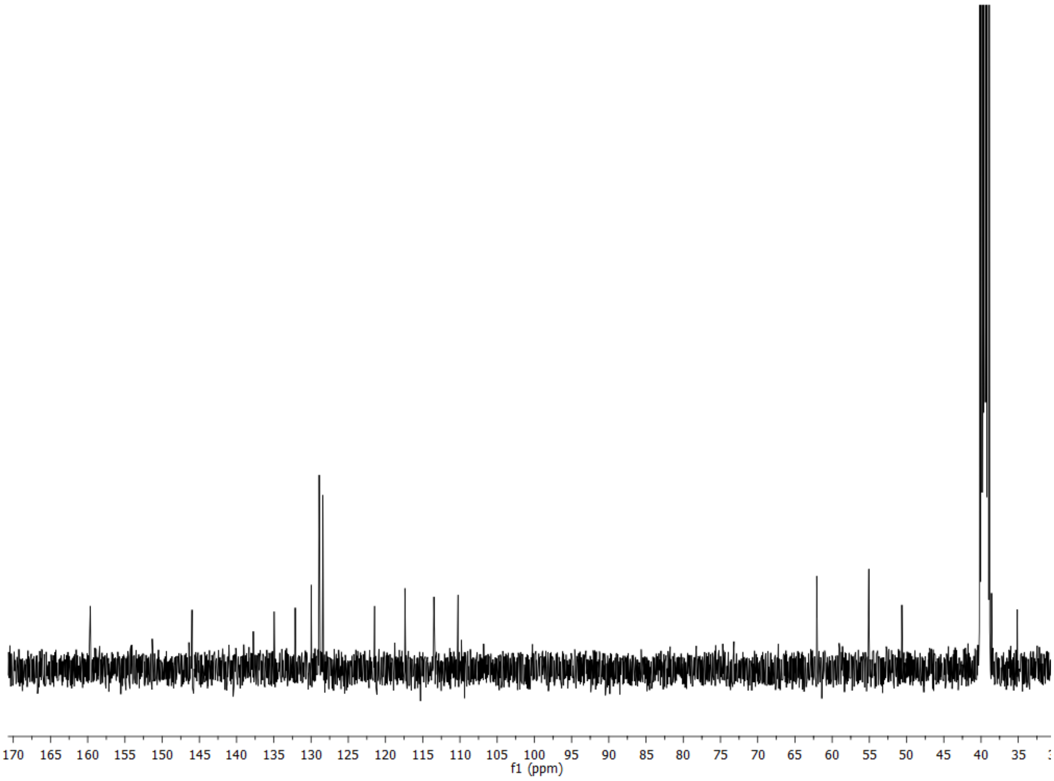
170 165 160 155 150 145 140 135 130 125 120 115 110 105 100 95 90 85 80 75 70 65 60 55 50 45 40 35
f1 (ppm)

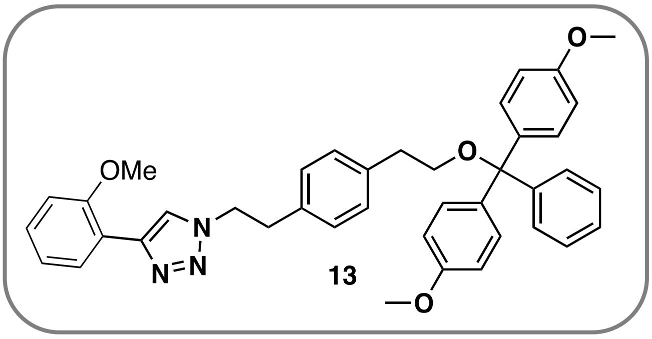
OMe
N
N
N
O
O
O
13

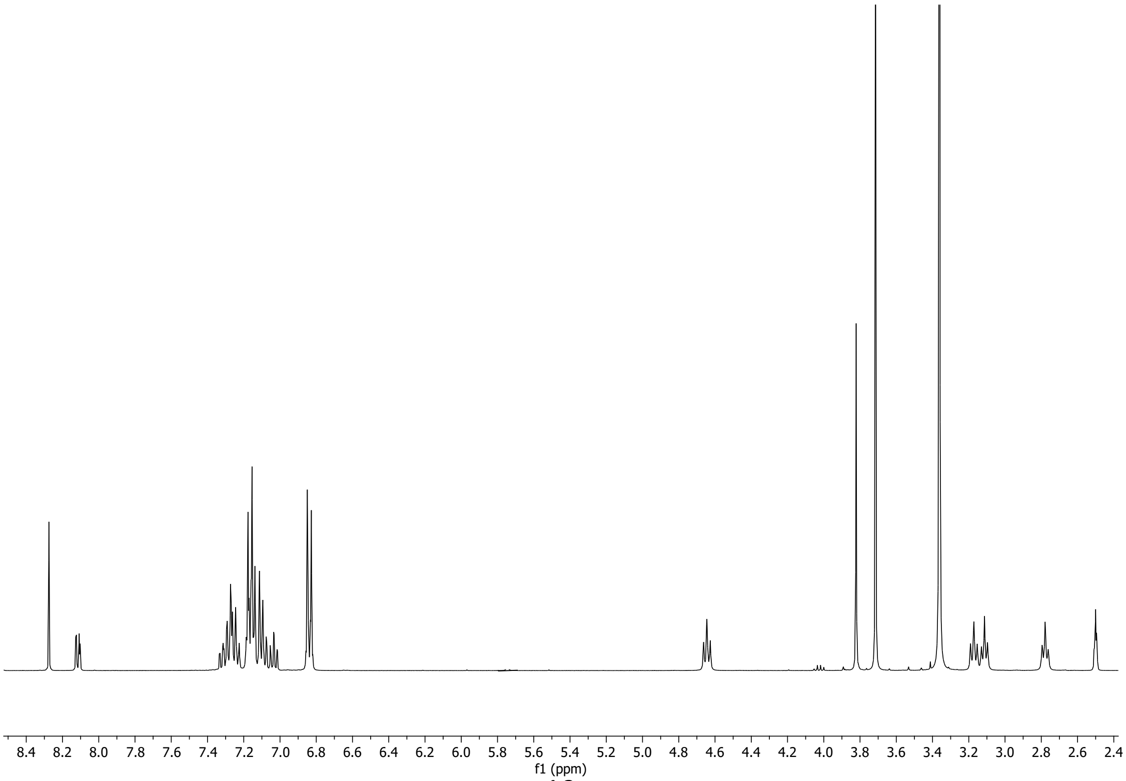
8.4 8.2 8.0 7.8 7.6 7.4 7.2 7.0 6.8 6.6 6.4 6.2 6.0 5.8 5.6 5.4 5.2 5.0 4.8 4.6 4.4 4.2 4.0 3.8 3.6 3.4 3.2 3.0 2.8 2.6 2.4
f1 (ppm)

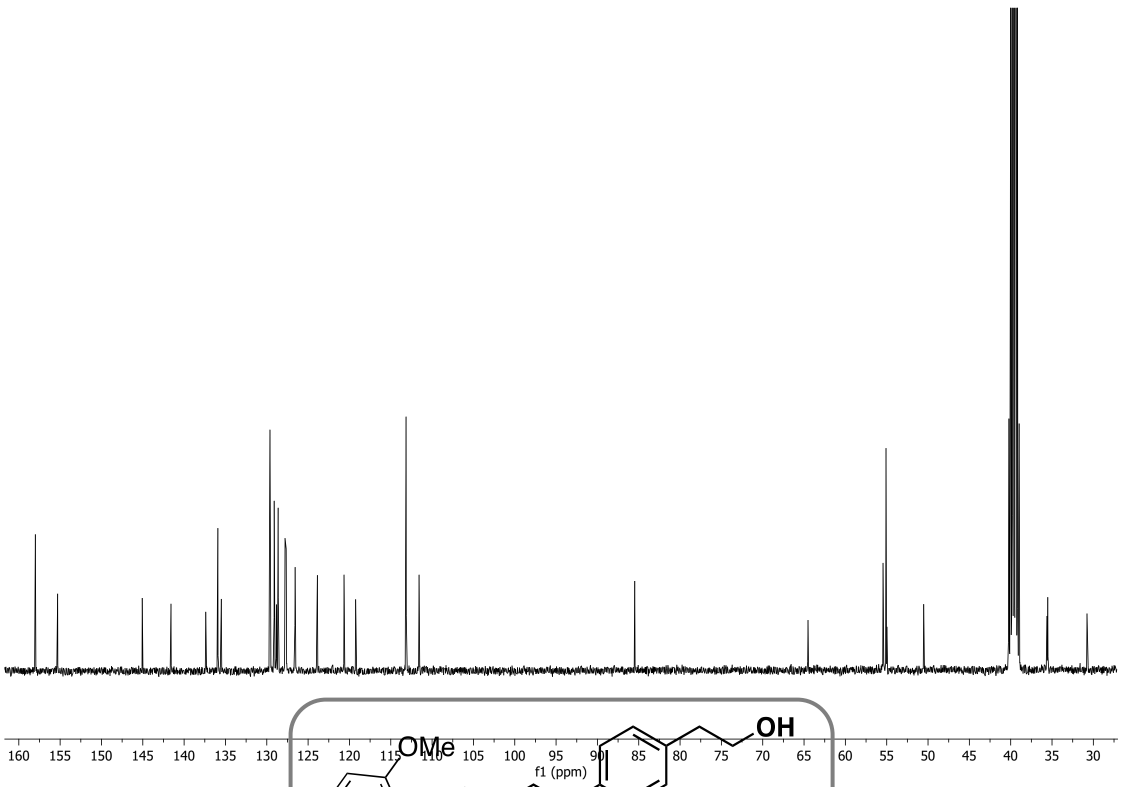
160 155 150 145 140 135 130 125 120 115 110 105 100 95 90 85 80 75 70 65 60 55 50 45 40 35 30
f1 (ppm)

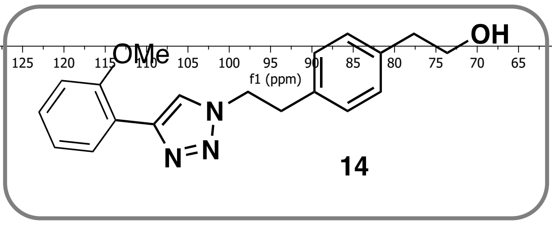
OMe
N
N
N
OH

14

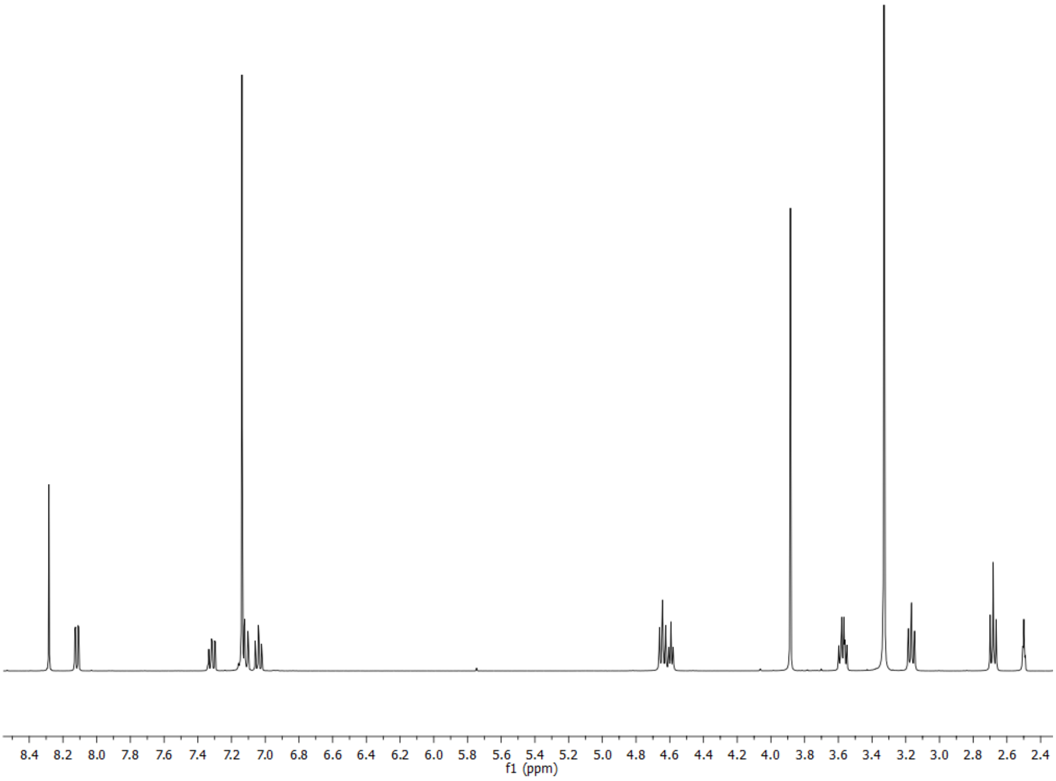
8.4 8.2 8.0 7.8 7.6 7.4 7.2 7.0 6.8 6.6 6.4 6.2 6.0 5.8 5.6 5.4 5.2 5.0 4.8 4.6 4.4 4.2 4.0 3.8 3.6 3.4 3.2 3.0 2.8 2.6 2.4
f1 (ppm)

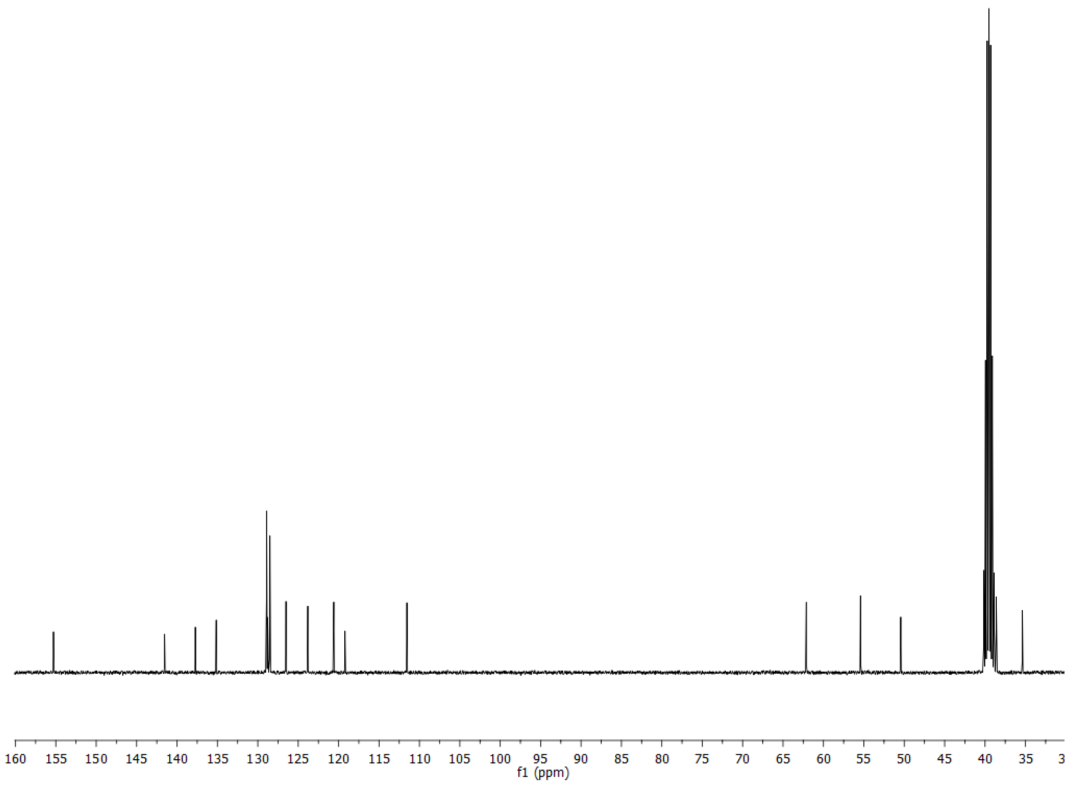
160 155 150 145 140 135 130 125 120 115 110 105 100 95 90 85 80 75 70 65 60 55 50 45 40 35 3
f1 (ppm)
